# Anti-Inflammatory Effects of Flavonoids in an LPS-Induced In Vitro Model of Canine Chronic Enteropathy

**DOI:** 10.3390/ani16030450

**Published:** 2026-02-01

**Authors:** Alma Virág Móritz, Nóra Luca Horváth, Rege Anna Márton, Anna Szilasi, Ákos Jerzsele, Roland Psáder, Orsolya Farkas

**Affiliations:** 1 Department of Pharmacology and Toxicology, University of Veterinary Medicine, 1078 Budapest, Hungary; cl9qv7@student.univet.hu (N.L.H.); jerzsele.akos@univet.hu (Á.J.); farkas.orsolya@univet.hu (O.F.); 2 National Laboratory of Infectious Animal Diseases, Antimicrobial Resistance, Veterinary Public Health and Food Chain Safety, University of Veterinary Medicine, 1078 Budapest, Hungary; 3 Department of Physiology and Biochemistry, University of Veterinary Medicine, 1078 Budapest, Hungary; marton.rege.anna@univet.hu; 4 Department of Pathology, University of Veterinary Medicine, 1078 Budapest, Hungary; szilasi.anna@univet.hu; 5 Department of Internal Medicine, University of Veterinary Medicine, 1078 Budapest, Hungary; psader.roland@univet.hu

**Keywords:** canine inflammatory enteropathy, lipopolysaccharide, oxidative stress, flavonoids, quercetin, luteolin, proanthocyanidins, in vitro model, cytokine modulation

## Abstract

Background: Chronic inflammatory enteropathies (CIEs) in dogs are multifactorial disorders characterized by mucosal immune dysregulation, compromised epithelial barrier function, and increased exposure to microbial components such as lipopolysaccharide (LPS). The resulting oxidative stress and inflammation contribute to local and systemic pathology. Objective: This study aimed to evaluate the immunomodulatory and antioxidant effects of three naturally occurring flavonoids—quercetin, luteolin, and grape seed extract oligomeric proanthocyanidins (GSOPs)—in LPS-stimulated canine duodenal explants. Methods: Duodenal tissue samples were cultured in vitro and challenged with LPS derived from *Escherichia coli* and *Salmonella enterica*. Explants were co-incubated with flavonoid compounds, and endpoints included evaluation of histological architecture, inflammatory cytokine production, and reactive oxygen species (ROS) and reactive nitrogen species (RNS) generation. Results: All three flavonoids attenuated LPS-induced mucosal inflammation and ROS production to varying degrees. In addition, GSOPs significantly reduced RNS levels under both basal and LPS-stimulated conditions. Quercetin and luteolin demonstrated pronounced downregulation of TNF-α, while both compounds also reduced IL-6 concentrations under non-stimulated conditions. These effects support the hypothesis that flavonoids can mitigate both inflammatory and oxidative responses under conditions relevant to CIE. Conclusion: Quercetin, luteolin, and GSOPs show promising in vitro efficacy in modulating key mechanisms implicated in canine CIE. Their multimodal actions highlight their potential as adjunctive nutraceuticals for the management of CIE in dogs. However, further in vivo validation is warranted.

## Simple Summary

Dogs with long-lasting intestinal problems often have an overactive immune response and a weakened gut barrier, which allows bacterial components to trigger inflammation and tissue damage. This study examined whether three natural plant compounds—quercetin, luteolin, and grape seed extract—can reduce these harmful reactions. Small sections of dog intestine were maintained in the laboratory and exposed to bacterial molecules that typically induce inflammation. When the plant compounds were added, all three helped reduce inflammatory signals and lowered the production of harmful oxidative molecules. Grape seed extract also decreased another type of reactive compound, even without an inflammatory trigger. Quercetin and luteolin were particularly effective in lowering key immune signals linked to chronic gut disease. These findings suggest that these natural substances may help support intestinal health in dogs by calming inflammation and reducing oxidative stress. Further studies in living animals are needed to confirm their potential benefits.

## 1. Introduction

Chronic inflammatory enteropathy (CIE) represents one of the most common causes of persistent gastrointestinal disease in dogs, manifesting clinically as chronic vomiting, diarrhea, and progressive weight loss. Rather than a single disease entity, CIE encompasses a spectrum of disorders that share similar clinical signs but differ substantially in their underlying mechanisms and therapeutic responsiveness. Following the exclusion of extraintestinal, infectious, parasitic, and neoplastic conditions, classification of CIE relies primarily on the dog’s response to targeted treatment strategies. This response-based framework reflects the biological heterogeneity of the condition and remains the most practical approach in both clinical and research settings. CIE subtypes include food-responsive enteropathy (FRE), food-responsive protein-losing enteropathy (FR-PLE), microbiota-related modulation-responsive enteropathy (MrMRE), immunosuppressant-responsive enteropathy (IRE), immunosuppressant-responsive protein-losing enteropathy (IR-PLE), non-responsive enteropathy (NRE), and non-responsive protein-losing enteropathy (NR-PLE). This response-based classification reflects the heterogeneous pathogenesis of CIE and is currently recommended for both clinical practice and research applications [1,2]. These subtypes often overlap in clinical presentation, and diagnosis typically relies on response-based classification, making differentiation challenging even with advanced diagnostic approaches such as endoscopy and histopathology [3]. The pathogenesis of canine CIE is complex and multifactorial. A prevailing hypothesis posits a dysregulated mucosal immune response to luminal antigens, including commensal bacteria, dietary components, and environmental triggers [2,4]. In FRE, adverse food reactions (AFRs) are thought to play a significant role, while in IRE and NRE, immune dysregulation predominates [2,5]. Genetic predispositions, including polymorphisms in genes encoding pattern recognition receptors (e.g., nucleotide-binding oligomerization domain 2 [NOD2] and Toll-like receptors [TLRs]), as well as alterations in the gut microbiome, have been implicated in canine CIE and share similarities with mechanisms described in human inflammatory bowel disease (IBD) [6,7].

Impairment of the intestinal epithelial barrier is a key pathological feature of canine CIE and represents a critical interface between luminal microbes and host immune responses. When barrier integrity is compromised, bacterial components that are normally restricted to the intestinal lumen, including lipopolysaccharide (LPS), gain access to the mucosal immune compartment. LPS is a potent innate immune stimulus that activates Toll-like receptor 4-dependent signaling pathways, leading to the initiation and amplification of inflammatory cascades [8]. In dogs affected by CIE, increased intestinal permeability has been associated with elevated circulating LPS levels, supporting the concept that microbial translocation contributes not only to local intestinal inflammation but also to systemic immune activation [4,9]. This phenomenon is supported by elevated circulating LPS concentrations in dogs with CIE, which correlate positively with disease activity scores and histopathological inflammation. Importantly, LPS translocation may not only drive local intestinal inflammation but also promote systemic low-grade inflammation, contributing to extraintestinal manifestations such as hepatic dysfunction and alterations in lipid metabolism—pathophysiological parallels also observed in human IBD and metabolic syndrome [10].

The structural heterogeneity of LPS molecules contributes to differences in their immunostimulatory properties. LPS derived from *Escherichia coli* and *Salmonella enterica* serovars, for example, can elicit variable cytokine and oxidative stress responses due to differences in their lipid A moieties and O-antigen side chains [11]. Such variability underscores the importance of selecting the LPS source in experimental models of mucosal inflammation. Furthermore, sustained LPS exposure has been shown to induce immune tolerance in some contexts while promoting hyperresponsiveness in others, depending on dose, duration, and cellular context. These dynamics add further complexity to the interpretation of LPS-mediated inflammation in both research and clinical settings [12].

Beyond immune activation driven by microbial products, oxidative and nitrosative stress substantially contribute to mucosal injury in canine CIE. During active inflammation, excessive generation of reactive oxygen species (ROS) by infiltrating immune cells disrupts epithelial integrity and reinforces pro-inflammatory signaling networks [13]. These redox-sensitive pathways create a self-sustaining loop in which oxidative damage and immune activation mutually amplify each other. Reactive nitrogen species (RNS), particularly nitric oxide and its downstream metabolites, further exacerbate epithelial dysfunction by promoting nitrosative modifications and impairing barrier function. Together, these processes delay mucosal repair and perpetuate chronic intestinal inflammation [14,15,16].

Given these multifactorial mechanisms, there is growing interest in therapies that not only suppress inflammation but also restore barrier integrity and redox balance. However, conventional treatments such as corticosteroids and immunosuppressants, while effective in many cases, carry risks of adverse effects, particularly with long-term use. Moreover, the efficacy of dietary management can vary, and antimicrobial therapy is no longer recommended as a routine treatment for CIE due to concerns regarding antimicrobial resistance and limited long-term benefit [1,2]. In this context, bioactive compounds derived from natural sources, particularly flavonoids, have gained attention for their pleiotropic therapeutic potential [17].

Flavonoids are bioactive polyphenolic compounds derived from plant sources and have attracted increasing interest as modulators of intestinal inflammation [18]. Their relevance in chronic gastrointestinal disorders stems from their capacity to influence multiple biological processes simultaneously, including redox balance, cytokine production, epithelial barrier stability, and host–microbe interactions [19]. Rather than acting through a single molecular target, flavonoids exert pleiotropic effects that depend on their chemical structure, local concentration, and cellular context. These characteristics make them particularly appealing as adjunctive agents in complex, multifactorial diseases such as canine CIE [20].

Quercetin is among the most extensively investigated flavonoids with regard to intestinal inflammation. Experimental studies have demonstrated that quercetin can attenuate pro-inflammatory signaling by interfering with transcriptional pathways involved in cytokine production, while simultaneously enhancing epithelial barrier resilience [21,22,23]. In addition to its direct antioxidant capacity, quercetin influences epithelial tight junction dynamics and modulates host responses to microbial stimuli. These combined properties suggest that quercetin may be particularly relevant in experimental systems that model barrier disruption and endotoxin-driven inflammation [24].

Luteolin is a naturally occurring flavonoid that has been reported to exert anti-inflammatory effects in various experimental models of intestinal inflammation [25]. Unlike flavonoids that primarily act as direct radical scavengers, luteolin has been reported to modulate intracellular signaling pathways involved in immune activation and epithelial stress responses [21]. Luteolin also interferes with inflammasome activation and has been demonstrated to reduce epithelial permeability and oxidative injury in models of colitis [26]. Furthermore, it exhibits selective antimicrobial activity against pathogenic bacteria while sparing commensals, suggesting a microbiota-modulatory role [27].

Grape seed oligomeric proanthocyanidins (GSOPs) represent a complex mixture of polyphenolic compounds with documented biological activity in inflammatory conditions [28]. Rather than targeting a single molecular pathway, GSOPs have been reported to influence redox homeostasis and inflammatory signaling in parallel. Their relevance in intestinal disease models lies in their capacity to limit oxidative damage while modulating host responses to inflammatory stimuli. In the context of chronic intestinal inflammation, such combined effects may contribute to improved epithelial resilience and attenuation of inflammation-associated tissue injury [29,30].

Despite these promising effects, clinical application of flavonoids faces significant challenges. Many flavonoids exhibit poor solubility, rapid metabolism, and limited gastrointestinal absorption, resulting in low systemic bioavailability. To overcome these limitations, novel delivery strategies such as nanoencapsulation, glycosylation, and conjugation with carrier molecules are being explored. High local concentrations, as achievable in ex vivo or in vitro systems, may also offer insights into their mechanistic actions, even if such levels are not readily attainable in vivo [31].

Given the ethical and logistical constraints of in vivo experimentation in companion animals, in vitro models—such as intestinal explant cultures—have emerged as valuable tools for studying mucosal immunology and therapeutic modulation. These systems preserve the complex architecture of the intestinal mucosa, including epithelial, immune, and stromal components, allowing for more physiologically relevant assessments of barrier function, cytokine production, and oxidative responses [32]. Moreover, explant models enable direct exposure to defined stimuli (e.g., LPS) and candidate compounds (e.g., flavonoids) under controlled conditions, thereby facilitating mechanistic insights that can inform future clinical applications [33].

The present study aimed to evaluate the anti-inflammatory and antioxidant effects of three selected flavonoids—quercetin, luteolin, and GSOPs—using a canine duodenal explant model stimulated with LPS derived from *E. coli* and *S. Enteritidis*. By assessing cytokine responses and ROS generation, the ability of these compounds to modulate key mechanisms implicated in canine CIE was characterized.

To our knowledge, this is the first study to directly compare the effects of quercetin, luteolin, and GSOPs in an LPS-stimulated canine duodenal explant system, including endotoxins from two distinct Gram-negative bacterial species. This approach provides novel translational insight into how different flavonoid classes may support mucosal homeostasis and guide the development of adjunctive nutraceutical strategies for dogs with chronic enteropathy.

## 2. Materials and Methods

### 2.1. Sample Collection

Duodenal biopsy specimens were obtained post mortem from an 11-year-old, neutered female mixed-breed dog with no known history or clinical signs of gastrointestinal disease. The animal was euthanized due to a pulmonary neoplasm unrelated to the gastrointestinal tract, and tissue collection was performed immediately after death with the owner’s informed consent. All procedures complied with international and Hungarian regulations, as well as institutional guidelines, and were approved by the Pest County Government Office, Department of Food Chain Safety, Plant and Soil Protection (permit no. PE/EA/00980-6/2022). Inflammation and oxidative stress were induced using *E. coli* O111:B4 LPS (Sigma-Aldrich, Darmstadt, Germany) and *S. Enteritidis* LPS (Sigma-Aldrich, Darmstadt, Germany). Test compounds included quercetin (≥95%, Sigma-Aldrich, Darmstadt, Germany), luteolin (≥98%, Sigma-Aldrich, Darmstadt, Germany), and purified GSOPs (≥98.8%, Sigma-Aldrich, St. Louis, MO, USA), selected for their known antioxidant and anti-inflammatory properties.

### 2.2. Duodenum Explant Isolation and Culture Conditions

Tissue isolation and incubation durations were adapted from previously published explant culture protocols [33]. After euthanasia, a distal 10–12 cm segment of the duodenum was excised. The tissue was washed thoroughly with phosphate-buffered saline (PBS, Gibco, Paisley, UK) supplemented with 5% penicillin-streptomycin (PenStrep, Lonza, Verviers, Belgium). Luminal contents were removed from the serosal side, and all subsequent samples were maintained on ice. A subset of tissue was fixed in 10% buffered formalin for histopathological confirmation of intestinal health. Under sterile conditions, the explants were rinsed again with PBS and PenStrep. Collagen-coated 96-well plates were loaded with 200 µL per well of culture medium composed of DMEM/F12 (Sigma-Aldrich, Darmstadt, Germany), 25 mL fetal bovine serum (FBS; EuroClone, Pero, Italy), 5 mL sodium glutamate (Sigma-Aldrich, Darmstadt, Germany), 5 mL PenStrep, and HCMTM SingleQuotsTM Kit (Lonza-Biocenter, Szeged, Hungary). Using a 1.5 mm biopsy punch (Rapid Core Punch, MDE Heidelberg), duodenal explants were placed into 80-well plates. The plates were incubated at 37 °C and 95% humidity with 5% CO_2_ for 3 h, after which the wells were washed with PBS [34].

Explants were then treated with LPS (from *E. coli* and *S. Enteritidis*) at 10 µg/mL to induce inflammation and oxidative stress. The 10 µg/mL concentration was chosen based on previous explant and epithelial-cell studies showing consistent cytokine and ROS induction at this dose [34]. Quercetin, luteolin, and GSOPs were applied at 25 and 50 µg/mL, according to prior concentration–effect data reported by our Department of Pharmacology and Toxicology. Flavonoids were dissolved in dimethyl sulfoxide (DMSO, Sigma-Aldrich, Darmstadt, Germany) at 1% *v*/*v*, and vehicle controls were included in all experiments; preliminary tests confirmed that this concentration of DMSO did not affect the measured outcomes. The experimental groups included a medium-only control, LPS alone, each flavonoid alone, and LPS combined with each flavonoid [35,36,37]. Following an additional 12 h incubation under the same conditions (37 °C, 5% CO_2_, 95% humidity), assays were performed to assess cell viability, ROS generation, and cytokine release.

### 2.3. Histopathological Analysis

Viability and histologic structure were assessed by routine histopathological evaluation. Samples from the 0-, 3-, 6-, 9-, and 12 h incubation periods were placed in 10% neutral buffered formalin for 12 h at room temperature, followed by paraffin embedding after a routine dehydration protocol. Sections 3–5 μm thick were trimmed from each specimen. Formalin-fixed, paraffin-embedded sections were stained with hematoxylin and eosin and examined using standard histological methods. Slides were scanned with a Pannoramic Midi II digital slide scanner (3DHistech, Budapest, Hungary), and representative images were captured using SlideViewer 2.6 software (3DHistech, Budapest, Hungary).

### 2.4. Metabolic Activity Assay and Cell Viability Assay

Metabolic activity was assessed using the CCK-8 assay kit (Cell Counting Kit-8, Sigma-Aldrich, Darmstadt, Germany). This colorimetric assay relies on the conversion of water-soluble tetrazolium salt WST-8 by metabolically active cells, and was performed following the manufacturer’s instructions. A 10 µL aliquot of CCK-8 reagent was added per well, and the plates were incubated for 1 h at 37 °C in 5% CO_2_. Absorbance was measured at 450 nm using a SpectraMax iD3 microplate reader (Molecular Devices, San Jose, CA, USA).

Cytotoxicity was determined by quantifying lactate dehydrogenase (LDH) released from damaged cells using a commercial LDH Activity Assay Kit (Sigma-Aldrich, Darmstadt, Germany). From each well, 10 µL of conditioned medium was collected and mixed with the freshly prepared reaction reagent according to the manufacturer’s protocol. LDH release occurs when cell membranes are compromised, which provides a sensitive indicator of cell damage. The reaction reagent supports the LDH-dependent conversion of lactate to pyruvate and the formation of a colored product detectable by spectrophotometry. Samples were incubated for 30 min at 37 °C, protected from light, and absorbance was measured at 450 nm using a microplate reader (SpectraMax iD3, Molecular Devices). Cytotoxicity was expressed relative to the untreated control.

### 2.5. Intracellular ROS Measurement and Extracellular Hydrogen Peroxide Quantification

Intracellular ROS were quantified using DCFH-DA (2′,7′-dichlorofluorescein diacetate; Sigma-Aldrich, Darmstadt, Germany). This non-fluorescent dye is deacetylated intracellularly and oxidized by ROS to yield fluorescent DCF. To protect the assay from light interference, all procedures were conducted under dark conditions. After washing, explants were incubated with DCFH-DA-containing medium for 1 h. Subsequently, supernatants were removed, and 70 µL of M-PER lysis buffer (Thermo Fisher Scientific Invitrogen, Waltham, MA, USA) was added to each well. Explants were sonicated for 20 s (3 pulses/sec) using a Bandelin Sonopuls HD 2200 homogenizer (Bandelin Electronic, Berlin, Germany) to ensure complete lysis. The lysates were centrifuged at 4 °C, 4500× *g* for 5 min. The supernatant (50 µL) was transferred to a black-walled, clear-bottom plate, and fluorescence was recorded using a SpectraMax iD3 at Ex 480 nm/Em 530 nm [38].

Hydrogen peroxide (H_2_O_2_) levels were determined using the Amplex™ Red hydrogen peroxide/peroxidase assay kit (Thermo Fisher Scientific Invitrogen, Waltham, MA, USA). In the presence of horseradish peroxidase (HRP), Amplex Red reagent reacts with H_2_O_2_ to form the fluorescent compound resorufin, allowing sensitive detection. A 10 mM Amplex Red stock solution was prepared in DMSO and stored at –20 °C in the dark. HRP was freshly prepared in PBS (pH 7.4) at a final concentration of 1 U/mL. Standard H_2_O_2_ solutions (0.1–10 µM) were made by serial dilution from a 1 mM stock. Fluorescence reactions were conducted in black-walled, clear-bottom 96-well plates in a final volume of 100 µL (PBS + 10 µM Amplex Red + 0.05 U HRP + sample or standard). After 30 min of incubation at room temperature in the dark, fluorescence was measured at Ex 530–560 nm/Em~590 nm. Background signals (reagent blanks and no-sample controls) were subtracted. H_2_O_2_ concentrations were calculated using a standard curve regression equation. The assay detection limit was in the nanomolar range, enabling sensitive quantification of low-level extracellular and cellular H_2_O_2_.

### 2.6. Extracellular RNS Measurement

NO levels were quantified using the Total Nitric Oxide Assay Kit (Thermo Fisher Scientific Invitrogen, Waltham, MA, USA). Due to the short half-life of NO, the assay measures its stable metabolites, nitrite (NO_2_^−^) and nitrate (NO_3_^−^), as indirect indicators of NO production. Samples were centrifuged at 10,000× *g* for 10 min at 4 °C to remove cellular debris. To quantify total NO, nitrate was enzymatically reduced to nitrite by nitrate reductase in the presence of NADPH as a cofactor. Specifically, samples were incubated with nitrate reductase and NADPH at 37 °C for 30 min to ensure complete conversion of nitrate to nitrite. Following enzymatic reduction, the resulting total nitrite concentration was determined via the Griess reaction. In this colorimetric assay, nitrite reacts with sulfanilamide under acidic conditions to form a diazonium salt, which subsequently couples with N-(1-naphthyl)-ethylenediamine dihydrochloride (NED) to produce a stable azo dye. The intensity of the resulting pink color, proportional to nitrite concentration, was measured spectrophotometrically at 540 nm using a SpectraMax iD3 microplate reader. Quantification was achieved by comparison to a standard curve generated with known concentrations of sodium nitrite ranging from 0 to 100 μM. The standards underwent the same enzymatic reduction and Griess reaction steps as the samples to ensure consistency.

### 2.7. TNF-α and IL-6 Quantification by ELISA

The inflammatory response was evaluated by measuring TNF-α and IL-6 levels in the explant culture supernatants. Canine TNF-α and IL-6 sandwich ELISA kits (ElabScience Bionovation Inc., Houston, TX, USA) were utilized according to the manufacturer’s protocols. Absorbance was measured at 450 nm using a SpectraMax iD3 microplate reader. Concentrations in pg/mL were derived using standard calibration curves.

### 2.8. Protein Quantification

To correct for variable cell numbers across samples, total protein content was measured using a BCA Protein Assay Kit (Thermo Fisher Scientific Invitrogen, Waltham, MA, USA), following the manufacturer’s instructions. BCA working reagent was prepared at a 50:1 ratio; bovine serum albumin (BSA) standards (0–2 mg/mL) were made in distilled water. Each lysate (10 µL) and standard was mixed with 200 µL of BCA working reagent in 96-well plates, shaken for 30 s, and incubated at 37 °C for 30 min. Absorbance at 562 nm was recorded using a SpectraMax iD3 reader. Protein concentrations were calculated from the standard curve and used to normalize cytokine measurements and other relevant assay results, thereby correcting for variability in tissue size and cell number across explants.

### 2.9. Statistical Analysis

Data analysis was performed using R software (version 3.3.2; R Foundation for Statistical Computing, Vienna, Austria). Prior to statistical testing, datasets were inspected for potential outliers, and the normality of residuals was assessed using the Shapiro–Wilk test. Homogeneity of variances among groups was verified with Levene’s test. Provided that these assumptions were met, differences between groups were analyzed using one-way analysis of variance (one-way ANOVA). When the ANOVA indicated a statistically significant overall effect, Tukey’s Honest Significant Difference (HSD) test was applied for post hoc pairwise comparisons. Statistical significance was defined as *p* ≤ 0.05. The data are presented as the mean ± standard deviation, and figures and results indicate the corresponding significance thresholds (e.g., *p* < 0.05, *p* < 0.01, *p* < 0.001). Quantitative differences are expressed as percentages or fold changes relative to the control condition where relevant. For all experiments, “*n*” denotes the number of independent duodenal explants derived from the same donor animal and cultured in separate wells. Explants within each group originated from distinct tissue punches to ensure independence at the tissue level. No biological replication across multiple animals was performed.

## 3. Results

### 3.1. Histopathological Analysis

Based on the histopathological evaluation, the epithelial layer, lamina propria, crypts, and submucosa were intact in samples examined immediately after biopsy collection. Degeneration of enterocytes and crypt epithelial cells increased progressively with longer incubation periods.

### 3.2. Metabolic Activity Assay and Viability (LDH Release) Assay

**Figure 1:**
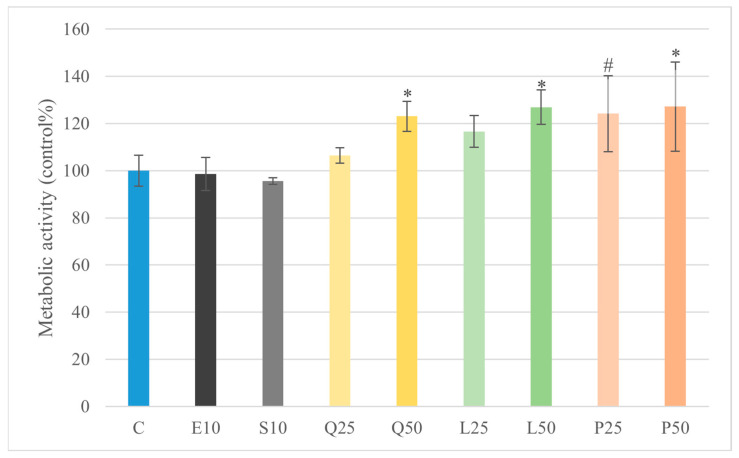
Investigation of LPS and flavonoid treatment on metabolic activity in duodenum explant culture with CCK-8 assay. Results are shown as mean values, along with their corresponding standard deviations. Mean of control group was set at 100% (*n* = 6/group). C: control; E10: *E. coli* LPS 10 μg/mL; S10: *S. Enteritidis* LPS 10 μg/mL; Q25, Q50: quercetin 25 and 50 μg/mL; L25, L50: luteolin 25 and 50 μg/mL; P25, P50: GSOPs 25 and 50 μg/mL. Trend toward significance: # *p* < 0.1; significant difference: * *p* < 0.05 compared to control group.

**Figure 2:**
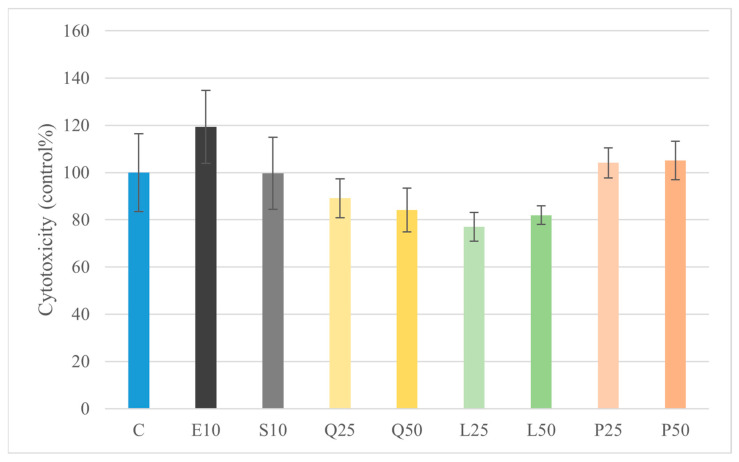
Investigation of LPS and flavonoid treatment on cell viability in duodenum explant culture with LDH activity assay. Results are shown as mean values, along with their corresponding standard deviations. Mean of control group set at 100% (*n* = 6/group). C: control; E10: *E. coli* LPS 10 μg/mL; S10: *S. Enteritidis* LPS 10 μg/mL; Q25, Q50: quercetin 25 and 50 μg/mL; L25, L50: luteolin 25 and 50 μg/mL; P25, P50: GSOPs 25 and 50 μg/mL.

Treatment with quercetin, luteolin, and GSOPs at a concentration of 50 µg/mL significantly increased metabolic activity (*p* < 0.05) of primary canine duodenal epithelial cells compared to the untreated control group, as assessed by the CCK-8 assay (Figure 1). Furthermore, none of the tested compounds induced significant cytotoxicity at the applied concentration, as indicated by the lack of elevated LDH activity in the culture supernatants compared to the control group (Figure 2). These results demonstrate that the quercetin, luteolin, GSOP, and LPS treatments were well tolerated by canine intestinal cells in vitro.

### 3.3. Intracellular ROS Measurement and Extracellular Hydrogen Peroxide Quantification

**Figure 3:**
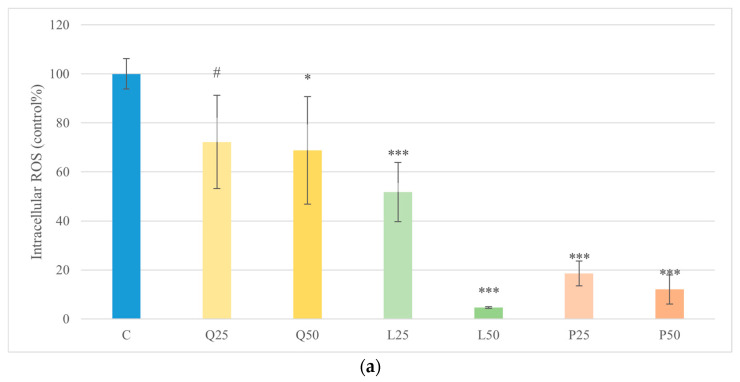

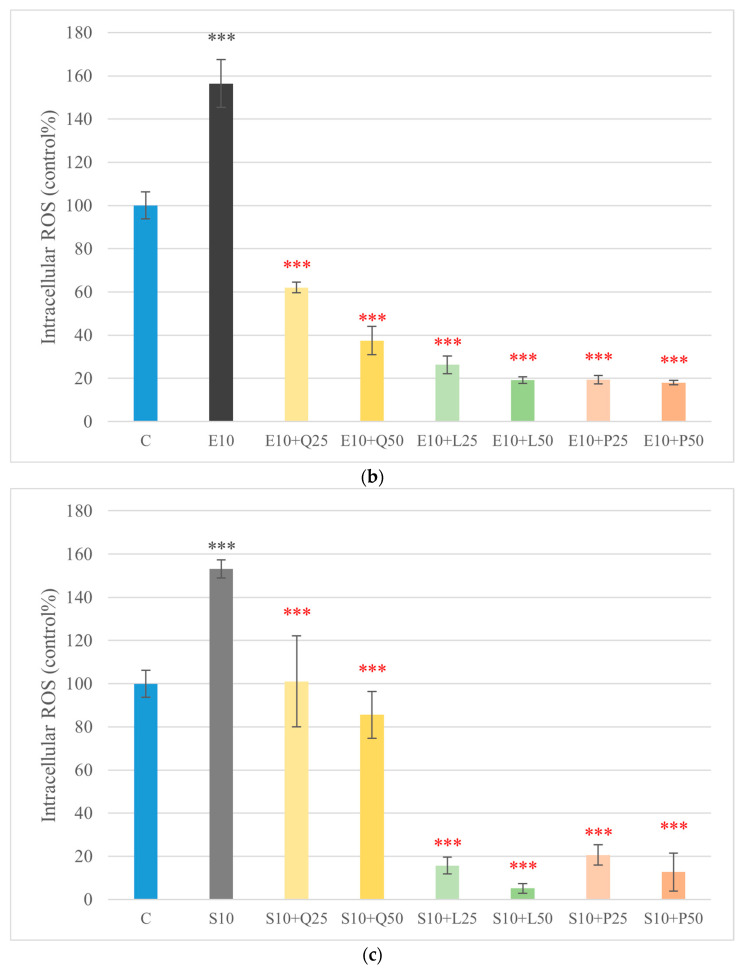
Effect of LPS and flavonoid treatment on intracellular ROS production in canine duodenum explant cultures with DCFH-DA probe. Results are shown as mean values, along with their corresponding standard deviations. Mean of control group set at 100% (*n* = 3/group). (**a**) Control and flavonoids alone: C (control); Q25/50 (quercetin 25/50 μg/mL); L25/50 (luteolin 25/50 μg/mL); P25/50 (GSOPs 25/50 μg/mL). (**b**) *E. coli* LPS ± flavonoids: E10 (LPS 10 μg/mL); E10+Q25/50, E10+L25/50, E10+P25/50. (**c**) *S. Enteritidis* LPS ± flavonoids: S10 (LPS 10 μg/mL); S10+Q25/50, S10+L25/50, S10+P25/50. Trend toward significance: # *p* < 0.1; significant difference: * *p* < 0.05; *** *p* < 0.001 compared to control group. *** *p* < 0.001 compared to corresponding LPS-treated groups.

Quercetin (50 μg/mL), luteolin (25 and 50 μg/mL), and GSOPs (25 and 50 μg/mL) significantly reduced basal ROS levels compared to the control group, while LPS derived from *E. coli* and *S. Enteritidis* in 10 μg/mL caused a significant increase (*p* < 0.001) in intracellular ROS level compared to the control group. Combined LPS and flavonoid treatments resulted in a significant decrease (*p* < 0.001) in intracellular ROS levels compared to the LPS treatment. The fluorescence intensity values were converted to control percentages (Figure 3).

**Figure 4:**
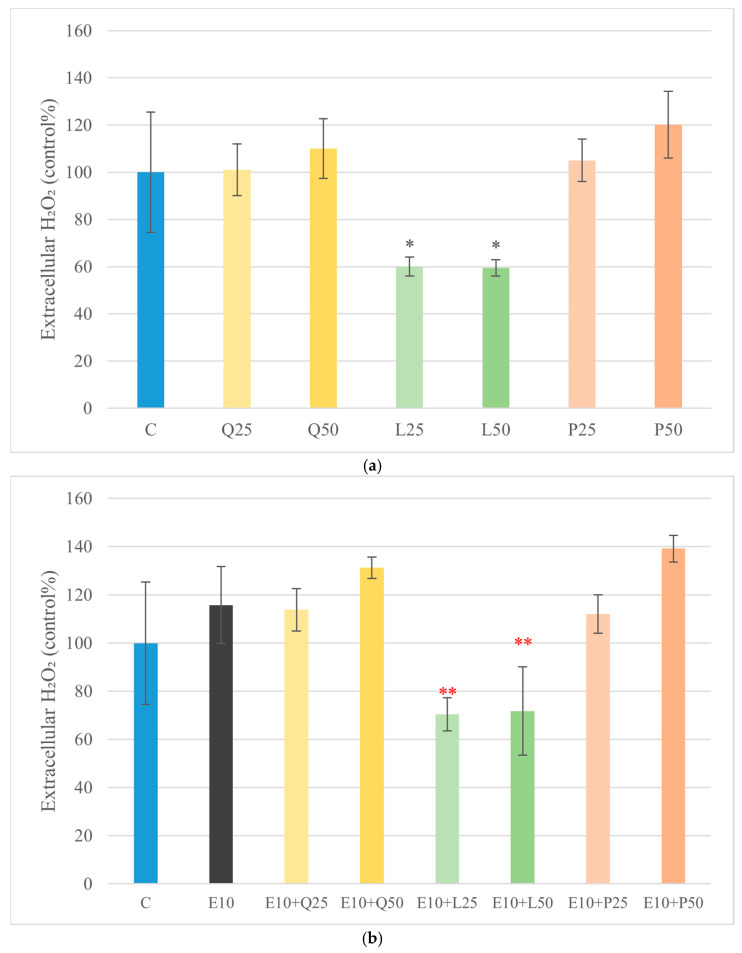

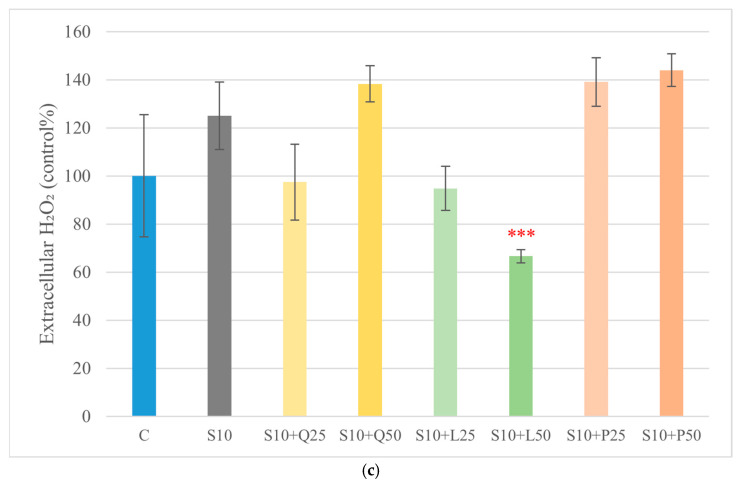
Effect of LPS and flavonoid treatment on extracellular H_2_O_2_ levels in canine duodenum explant cultures measured by Amplex Red probe. Results are shown as mean values, along with their corresponding standard deviations. Mean of control group set at 100% (*n* = 3/group). (**a**) Control and flavonoids alone: C (control); Q25/50 (quercetin 25/50 μg/mL); L25/50 (luteolin 25/50 μg/mL); P25/50 (GSOPs 25/50 μg/mL). (**b**) *E. coli* LPS ± flavonoids: E10 (LPS 10 μg/mL); E10+Q25/50, E10+L25/50, E10+P25/50. (**c**) *S. Enteritidis* LPS ± flavonoids: S10 (LPS 10 μg/mL); S10+Q25/50, S10+L25/50, S10+P25/50. Significant difference: * *p* < 0.05 compared to control group. ** *p* < 0.01; *** *p* < 0.001 compared to corresponding LPS-treated groups.

Hydrogen peroxide (H_2_O_2_) release into the culture medium was measured to assess oxidative stress after treatment with LPS and flavonoids, either alone or in combination. Luteolin (25 and 50 μg/mL) significantly reduced (*p* < 0.05) basal H_2_O_2_ levels, while quercetin and GSOPs did not differ notably from the control. LPS treatment alone (E10 and S10) did not elevate H_2_O_2_ levels with statistical significance. However, co-treatment of *E. coli* LPS with luteolin led to a significant decrease (*p* < 0.01) in extracellular H_2_O_2_ levels. A more pronounced reduction was observed (*p* < 0.001) in the case of *S. Enteritidis* LPS combined with the luteolin-treated group (Figure 4).

### 3.4. Extracellular RNS Measurement

**Figure 5:**
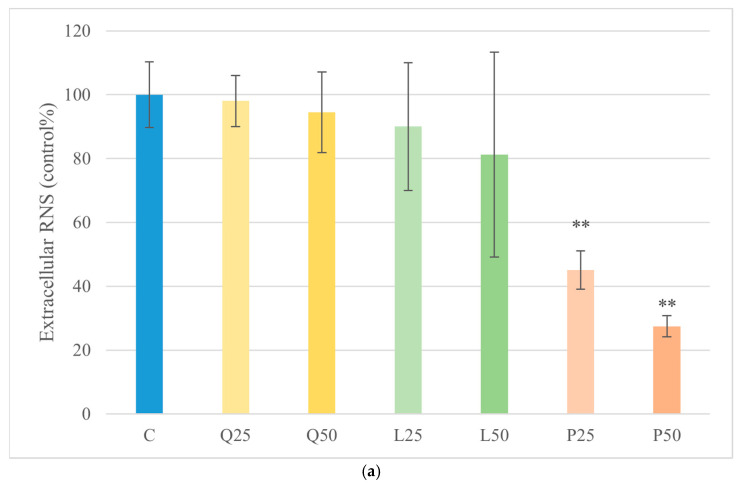

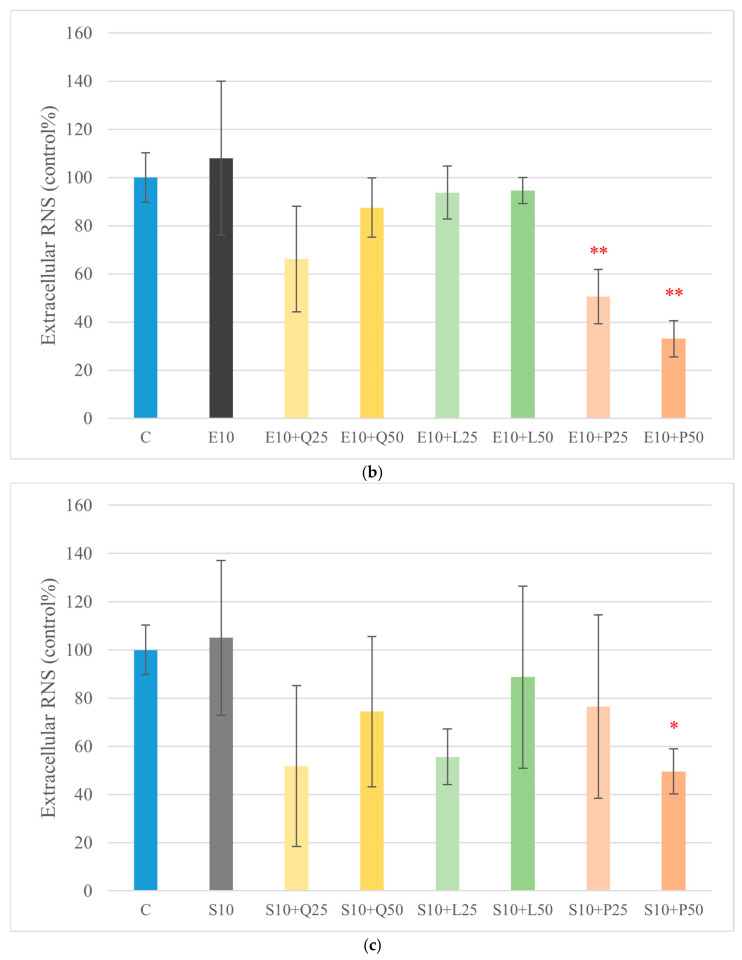
Effect of LPS and flavonoid treatment on extracellular RNS levels in canine duodenum explant cultures measured by Griess reaction. Results are shown as mean values, along with their corresponding standard deviations. Mean of control group set at 100% (*n*  =  3/group). (**a**) Control and flavonoids alone: C (control); Q25/50 (quercetin 25/50 μg/mL); L25/50 (luteolin 25/50 μg/mL); P25/50 (GSOPs 25/50 μg/mL). (**b**) *E. coli* LPS ± flavonoids: E10 (LPS 10 μg/mL); E10+Q25/50, E10+L25/50, E10+P25/50. (**c**) *S. Enteritidis* LPS ± flavonoids: S10 (LPS 10 μg/mL); S10+Q25/50, S10+L25/50, S10+P25/50. Significant difference: ** *p* < 0.01 compared to control group. * *p* < 0.05; ** *p* < 0.01 compared to corresponding LPS-treated groups.

NO levels, as an indicator of RNS production, were assessed following treatment. GSOPs at 25 and 50 µg/mL significantly reduced (*p* < 0.01) RNS levels compared with the untreated control. Under *E. coli* LPS stimulation, GSOP treatment at both 25 µg/mL and 50 µg/mL resulted in a significant decrease (*p* < 0.01) in RNS levels relative to the corresponding LPS-treated groups. In contrast, in *S. Enteritidis* LPS-stimulated explants, only the highest concentration (50 µg/mL) of GSOPs significantly lowered *(p* < 0.05) RNS levels compared with the respective LPS-treated controls (Figure 5).

### 3.5. TNF-α and IL-6 Quantification by ELISA

**Figure 6:**
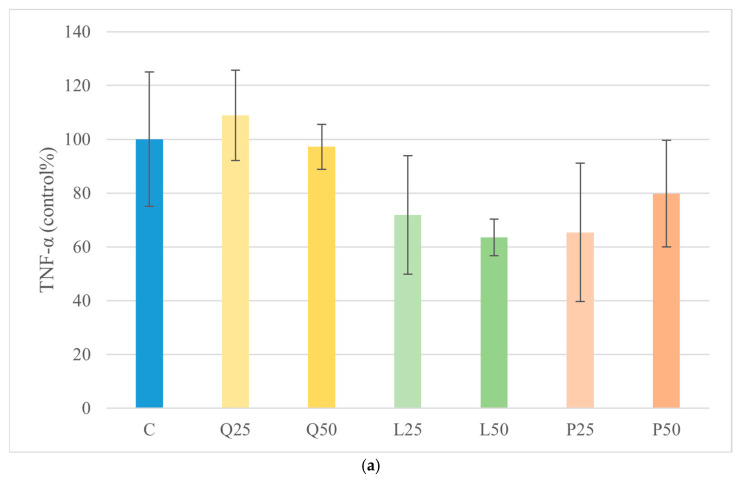

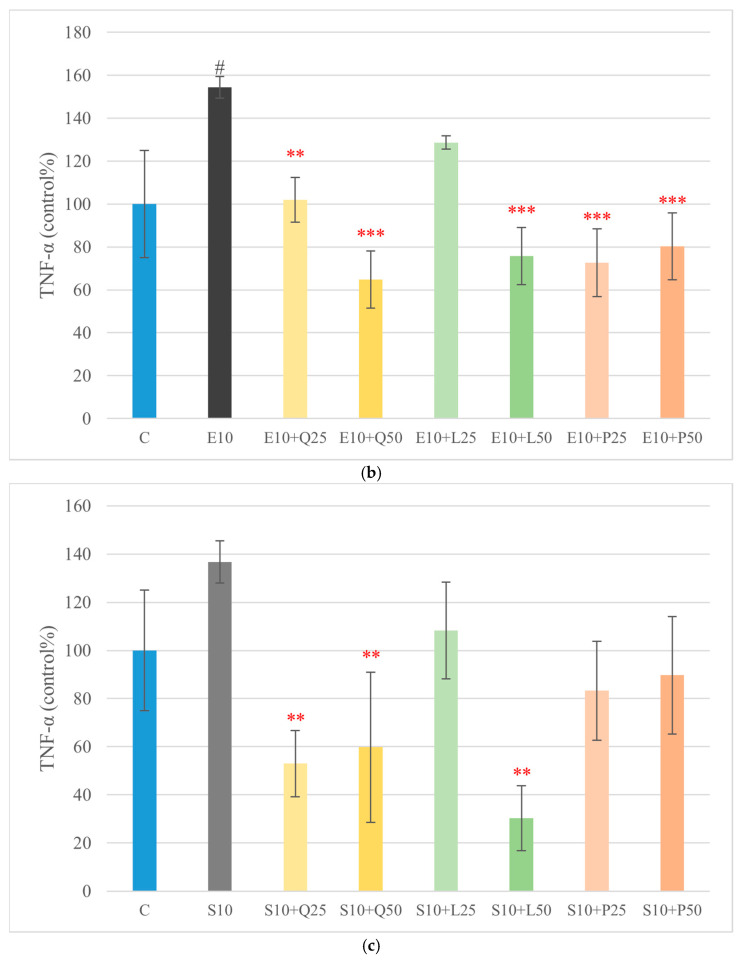
Effect of LPS and flavonoid treatment on TNF-α production in canine duodenum explant cultures. Results are shown as mean values, along with their corresponding standard deviations. Mean of control group set at 100% (*n* = 3/group). (**a**) Control and flavonoids alone: C (control); Q25/50 (quercetin 25/50 μg/mL); L25/50 (luteolin 25/50 μg/mL); P25/50 (GSOPs 25/50 μg/mL). (**b**) *E. coli* LPS ± flavonoids: E10 (LPS 10 μg/mL); E10+Q25/50, E10+L25/50, E10+P25/50. (**c**) *S. Enteritidis* LPS ± flavonoids: S10 (LPS 10 μg/mL); S10+Q25/50, S10+L25/50, S10+P25/50. Trend toward significance: # *p* < 0.1; significant differences compared to control group. ** *p* < 0.01, *** *p* < 0.001 compared to corresponding LPS-treated groups.

**Figure 7:**
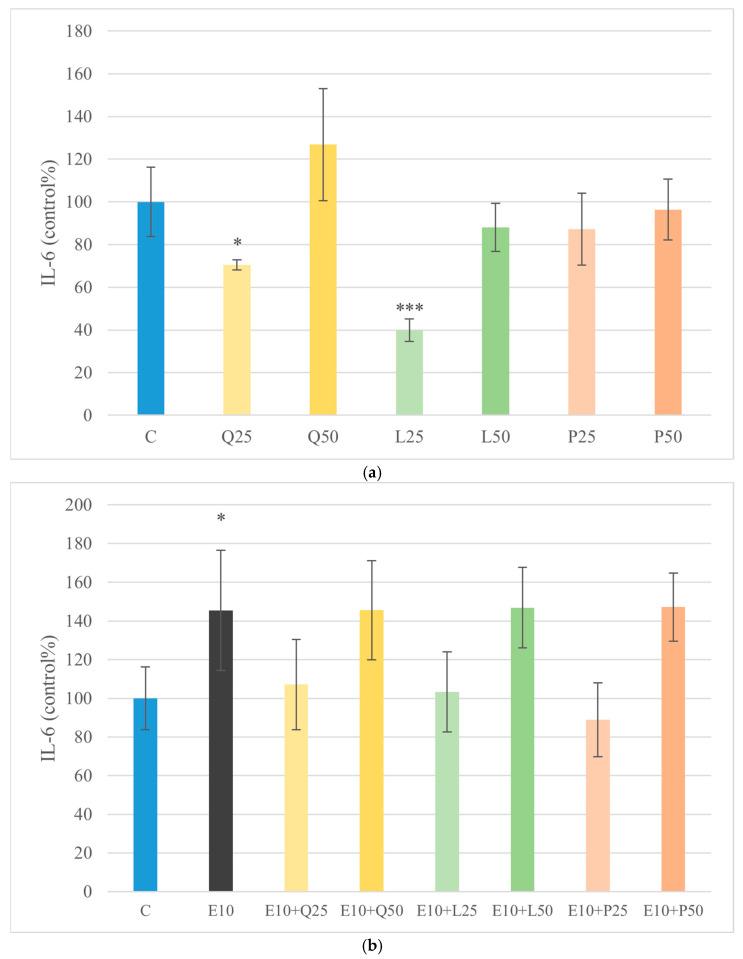

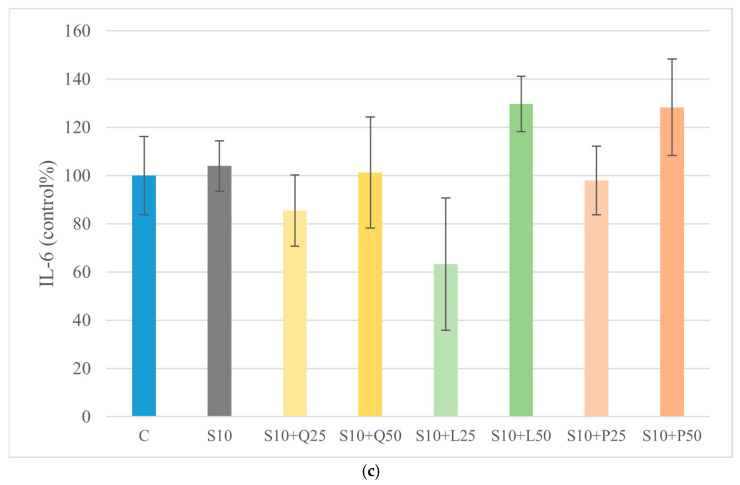
Effect of LPS and flavonoid treatment on IL-6 production in canine duodenum explant cultures. Results are shown as mean values, along with their corresponding standard deviations. Mean of control group set at 100% (*n* = 3/group). (**a**) Control and flavonoids alone: C (control); Q25/50 (quercetin 25/50 μg/mL); L25/50 (luteolin 25/50 μg/mL); P25/50 (GSOPs 25/50 μg/mL). (**b**) *E. coli* LPS ± flavonoids: E10 (LPS 10 μg/mL); E10+Q25/50, E10+L25/50, E10+P25/50. (**c**) *S. Enteritidis* LPS ± flavonoids: S10 (LPS 10 μg/mL); S10+Q25/50, S10+L25/50, S10+P25/50. Significant differences: * *p* < 0.05, *** *p* < 0.001 compared to control group.

TNF-α levels were measured to evaluate the pro-inflammatory response of canine duodenal explants after treatment with LPS and flavonoids, alone or in combination. Quercetin, luteolin, and GSOPs alone did not significantly alter TNF-α levels compared to the untreated control. Stimulation with *E. coli* LPS increased TNF-α production, indicating an inflammatory response. However, co-treatment with all three flavonoids, particularly at a concentration of 50 µg/mL, significantly attenuated LPS-induced TNF-α release (*p* < 0.001). Similarly, *S. Enteritidis* LPS caused a moderate but not statistically significant increase in TNF-α secretion. In groups co-treated with quercetin and luteolin, TNF-α levels were significantly reduced compared to the *S. Enteritidis* LPS-treated group (*p* < 0.01), demonstrating their anti-inflammatory potential under endotoxin-induced stress conditions (Figure 6). To further assess the inflammatory profile of the explants under these experimental conditions, IL-6 production was also quantified. Both quercetin and luteolin at 25 µg/mL significantly decreased (*p* < 0.05; *p* < 0.01) IL-6 levels compared with the untreated control group. Treatment with *E. coli* LPS significantly increased IL-6 production (*p* < 0.05) relative to the control. However, in LPS-stimulated explants, none of the flavonoid treatments produced significant changes in IL-6 concentrations when compared with their respective LPS-treated groups. Importantly, flavonoid co-treatment attenuated LPS-induced TNF-α release, whereas none of the tested compounds significantly reduced the LPS-driven increase in IL-6, indicating a cytokine-specific difference in responsiveness under endotoxin stimulation (Figure 6 and Figure 7). Moreover, direct comparison of the two LPS-only groups revealed that *E. coli* LPS induced significantly higher IL-6 production than *S. Enteritidis* LPS (*p* < 0.05), indicating endotoxin source-dependent differences in IL-6 induction.

## 4. Discussion

CIE is currently classified according to therapeutic response, reflecting its heterogeneous pathogenesis. Dietary intervention remains the cornerstone of management, particularly in food-responsive enteropathies; however, sustained remission often requires long-term adherence, and a proportion of dogs fail to respond adequately or experience relapse. In cases refractory to dietary management, treatment escalation is typically directed toward immunomodulatory or immunosuppressive therapies, as routine antibiotic use is no longer recommended in the management of CIE due to limited long-term efficacy and concerns regarding antimicrobial resistance and microbiota disruption [1,2]. Within this contemporary therapeutic framework, nutraceuticals should not be considered substitutes for established treatment strategies, but rather, as adjunctive interventions that may support dietary or immunosuppressive approaches. By modulating intestinal barrier integrity, immune responses, or microbial homeostasis, nutraceuticals may contribute to improved disease control or help reduce treatment intensity in selected cases [3,6]. Nevertheless, their role remains supportive, and well-designed controlled studies are needed to better define their clinical utility across different CIE subtypes.

In the present study, a canine duodenal explant model was used to examine how selected flavonoids influence inflammatory and oxidative responses under endotoxin challenge. By preserving the native mucosal architecture, this experimental system allowed assessment of epithelial and immune interactions that are directly relevant to the pathophysiology of canine CIE [39]. Exposure to LPS derived from two distinct Gram-negative bacterial sources provided a controlled inflammatory stimulus, enabling evaluation of compound-specific effects on cytokine release and redox balance. LPS from *E. coli* and *S. Enteritidis* was used to mimic bacterial stimuli, as both pathogens are common members of the canine gut microbiota and play a central role in CIE pathogenesis through their endotoxins. The use of ex vivo cultured duodenal biopsy tissues has been validated as a model to study mucosal immune responses and cytokine expression in dogs with chronic enteropathy, demonstrating that duodenal explants can respond to pathogen-associated molecular patterns via TLR signaling and cytokine modulation, faithfully recapitulating in vivo inflammation patterns [40,41]. These findings support the hypothesis that flavonoids can attenuate key pathogenic mechanisms implicated in canine CIE, namely cytokine-mediated inflammation and oxidative stress. A central element of CIE pathogenesis is the breakdown of intestinal barrier integrity, which facilitates the translocation of microbial components such as LPS into the lamina propria and, subsequently, systemic circulation. Elevated circulating LPS levels have been correlated with clinical disease severity in dogs with CIE and may serve as a surrogate marker of mucosal barrier dysfunction [42,43]. In this context, our model simulates critical aspects of CIE pathophysiology, particularly LPS-induced epithelial inflammation.

In our explant model, treatment of canine duodenal epithelial cells with quercetin, luteolin, and GSOPs at a concentration of 50 µg/mL resulted in a significant increase in metabolic activity as measured by the CCK-8 assay (*p* < 0.05), indicating enhanced cell viability and general proliferation compared to untreated controls. Importantly, no compound induced significant cytotoxicity at this concentration, as indicated by unchanged LDH release in culture supernatants. These results confirm that the flavonoids, at the tested concentrations, were well tolerated by primary canine intestinal explant cultures. [44]. Correspondingly, all three flavonoids—quercetin (50 µg/mL), luteolin (25 and 50 µg/mL), and GSOPs (25 and 50 µg/mL)—significantly reduced both basal and LPS-induced intracellular ROS levels in explant cultures (*p* < 0.001). LPS alone (10 µg/mL from *E. coli* or *S. Enteritidis*) markedly increased ROS production, while flavonoid co-treatment reversed this effect to below control levels. These findings are in line with earlier in vitro studies demonstrating that quercetin and luteolin significantly reduce intracellular oxidative stress in various LPS-stimulated immune cell cultures by scavenging free radicals and enhancing antioxidant enzyme activity [37]. At first glance, the observation that LPS stimulation significantly increased intracellular ROS levels without inducing a corresponding increase in extracellular H_2_O_2_ may appear contradictory. However, this pattern reflects fundamental differences in the oxidative compartments and reactive species detected by the applied assays. The DCFH-DA probe primarily measures intracellular ROS generated within epithelial and immune cells, including H_2_O_2_ and other redox-active intermediates involved in inflammatory signaling. In contrast, the Amplex Red assay selectively detects extracellular H_2_O_2_ released into the culture medium. Accordingly, enhanced intracellular ROS generation following LPS exposure does not necessarily translate into proportional extracellular H_2_O_2_ release. Efficient intracellular antioxidant buffering, rapid enzymatic detoxification, or limited diffusion of H_2_O_2_ across cellular membranes may further contribute to this dissociation. These findings highlight that intracellular and extracellular oxidative readouts represent complementary but distinct aspects of oxidative stress and should be interpreted accordingly rather than as directly interchangeable endpoints [45]. Polyphenolic flavonoids, such as quercetin, luteolin, and GSOPs, frequently used as nutraceuticals, have been shown to downregulate iNOS/NO output in LPS-stimulated macrophage/epithelial models via NF-κB/MAPK modulation, with inhibition verified by Griess readouts. Collectively, these data suggest that targeting nitrosative stress with dietary polyphenols could complement strategies aimed at resolving oxidative stress and restoring epithelial integrity in canine CIE [46,47,48].

Moreover, flavonoid treatments in the presence of LPS significantly attenuated the release of TNF-α, as measured by ELISA. Quercetin and GSOPs at 25 and 50 µg/mL both caused a robust reduction in TNF-α secretion (*p* < 0.001), whereas luteolin only at 25 µg/mL reduced it—consistent with dose-dependent and structural variability in immune responses. These cytokine-modulatory effects are consistent with the proposed mechanisms of NF-κB and MAPK pathway inhibition by quercetin and luteolin, reducing pro-inflammatory mediator release in endotoxin-challenged cells [49]. Importantly, this observation is highly relevant to the pathophysiology of canine CIE, in which TNF-α is considered a central pro-inflammatory mediator contributing to intestinal immune activation and tissue damage [41]. Therefore, the marked attenuation of LPS-induced TNF-α secretion by quercetin and luteolin in canine duodenal explants supports their potential as dietary immunomodulatory compounds, with translational relevance for limiting TNF-α-driven inflammation in CIE. Although extrapolation to in vivo disease requires further investigation, these findings highlight a clinically meaningful cytokine target that may be responsive to flavonoid intervention. In parallel, IL-6 production was quantified to further characterize the inflammatory profile of the explants. Quercetin and luteolin at 25 µg/mL significantly decreased (quercetin: *p* < 0.05; luteolin: *p* < 0.01) IL-6 levels compared with the untreated control group, indicating basal anti-inflammatory activity in the absence of endotoxin challenge. As expected, exposure to *E. coli* LPS significantly increased (*p* < 0.05) IL-6 concentrations relative to the control. However, in LPS-stimulated explants, none of the flavonoid treatments produced significant changes in IL-6 compared with their respective LPS-treated controls. Interestingly, IL-6 induction differed significantly between the two endotoxins, as *E. coli* LPS elicited a stronger IL-6 response than *S. Enteritidis* LPS, suggesting that IL-6 regulation in this model is sensitive not only to the presence of endotoxin stimulation but also to the specific LPS source and its qualitative signaling properties. Notably, quercetin and luteolin therefore reduced IL-6 under basal conditions but failed to significantly suppress LPS-induced IL-6, in contrast to their robust inhibition of TNF-α. This discrepancy suggests pathway-specific regulation of cytokine responses rather than a uniform anti-inflammatory effect across all pro-inflammatory mediators. Although NF-κB activation is a major upstream driver of both TNF-α and IL-6 transcription in response to LPS, IL-6 expression can additionally be sustained by signaling circuits involving STAT3 and MAPK pathways, which may remain active or become compensatory under strong endotoxin stimulation. In such conditions, flavonoid-mediated attenuation of NF-κB may be sufficient to blunt TNF-α release, whereas IL-6 production may persist due to redundant or temporally distinct signaling inputs that maintain IL-6 transcription and secretion even when NF-κB activity is partially reduced. These findings indicate that IL-6 is less responsive to flavonoid-mediated modulation under strong LPS stimulation than TNF-α, potentially reflecting differential dependence on NF-κB versus alternative downstream signaling nodes. This pattern aligns with studies showing that quercetin and luteolin suppress IL-6 expression in LPS-activated immune cells under moderate stimulation, yet are less effective once inflammatory cascades are strongly induced. For example, in murine RAW 264.7 macrophages, quercetin markedly reduced IL-6 secretion, as demonstrated using ELISA [50]. Similarly, luteolin inhibited LPS-induced IL-6 gene expression and release in RAW 264.7 and MH-S cells via suppression of NF-κB and MAPK signaling [51,52,53]. Notably, flavonoids such as quercetin also interact with cytoprotective pathways beyond NF-κB inhibition, including activation of nuclear factor erythroid 2-related factor 2 (Nrf2), which modulates oxidative stress and inflammatory signaling in epithelial tissues. In line with this, quercetin was shown to alleviate deoxynivalenol-induced intestinal barrier dysfunction in IPEC-J2 cells and weaned piglets through activation of Nrf2 signaling, highlighting that the anti-inflammatory impact of quercetin may depend on broader homeostatic mechanisms linked to oxidative balance and epithelial resilience rather than direct suppression of all cytokines under maximal endotoxin challenge [54]. In addition, the intestinal explant model contains multiple cellular compartments, and TNF-α and IL-6 may originate from partially distinct cell populations. TNF-α is typically produced rapidly and predominantly by activated myeloid cells, whereas IL-6 can be sustained by several cell types, including epithelial and stromal cells, potentially contributing to different kinetics and differential sensitivity to flavonoid-mediated pathway modulation [55]. Taken together, the ability to suppress TNF-α more effectively than IL-6 suggests that these flavonoids may preferentially dampen acute TNF-α-dominated inflammatory signaling, which is a relevant component of the cytokine milieu in canine CIE. Consequently, the present results support the concept that dietary polyphenols may help mitigate low-grade nitrosative and cytokine-driven inflammation in CIE, although their efficacy may vary depending on cytokine-specific signaling regulation, stimulus intensity, and temporal dynamics of pathway activation. While the present study was conducted using an in vitro explant model, limited in vivo data are available regarding the effects of polyphenolic compounds in dogs with chronic inflammatory conditions. Notably, supplementation with GSOPs has been shown to reduce intestinal inflammation and modulate gut microbiota composition in dogs with mild CIE, supporting a potential adjunctive role for these compounds [56]. In addition, dietary quercetin supplementation has been investigated in canine feeding trials and was found to influence metabolic and inflammatory pathways without adverse effects [57].

Importantly, the flavonoid concentrations applied in the present in vitro experiments (25–50 µg/mL) are not intended to directly correspond to achievable systemic tissue concentrations following oral administration. In vivo, flavonoids such as quercetin undergo extensive digestion, microbial transformation, and hepatic metabolism, resulting in limited systemic bioavailability in dogs [58]. However, following oral supplementation, substantially higher transient concentrations may be achieved locally within the intestinal lumen, particularly before absorption and metabolism, suggesting that luminal exposure of the intestinal mucosa to flavonoids could reach levels relevant for local epithelial and immune modulation. Accordingly, the effects observed in this study should be interpreted as mechanistic proof-of-concept demonstrating the capacity of these compounds to modulate oxidative stress and inflammatory signaling at the mucosal level, rather than as a basis for direct dose extrapolation or clinical efficacy claims. Together, these observations highlight the need for controlled pharmacokinetic studies assessing luminal and tissue exposure, as well as well-designed clinical trials, to determine whether the anti-inflammatory and antioxidant effects demonstrated in vitro translate into clinically meaningful benefits in dogs with CIE.

The explant model applied in this study offers a physiologically relevant platform for investigating mucosal immune responses under controlled conditions. By preserving native epithelial, stromal, and immune structures, it enables the assessment of complex inflammatory interactions that cannot be reproduced in monoculture systems. The use of LPS from two distinct Gram-negative species further strengthens the translational value of the model by reflecting the heterogeneity of bacterial stimuli encountered in canine chronic enteropathy. At the same time, several methodological limitations should be considered when interpreting the present findings. As is inherent to ex vivo intestinal explant models, tissue integrity gradually declined during prolonged incubation, which was confirmed by histopathological evaluation showing a time-dependent reduction in epithelial viability. Accordingly, histological analysis was restricted to descriptive assessment of epithelial architecture and overall tissue viability. Given the progressive degeneration of explants over time and the use of tissue derived from a single donor animal, semi-quantitative histological scoring was not applied, as such scoring would not reliably distinguish treatment-related effects from incubation-associated tissue deterioration. Instead, histopathology was used to support the interpretation of functional outcome measures, including cytokine production and oxidative stress markers.

Importantly, all explants used in this study originated from a single clinically healthy donor dog. Wells therefore represent technical replicates only, generated from independent tissue punches of the same animal, and no biological replication across multiple individuals was performed. Consequently, inter-individual variability could not be assessed, and the findings should be regarded as exploratory and hypothesis-generating rather than broadly generalizable. The results obtained at later incubation time points likely reflect a combination of treatment effects and the natural decline in explant viability. In addition, some outcome measures were assessed with a limited number of explants per group (*n* = 3), which inherently restricts statistical power. Although this sample size is commonly used in technically demanding explant models, non-significant or marginal findings should be interpreted with caution, as the absence of statistical significance does not necessarily indicate a lack of biological effect. Furthermore, the explant system lacks systemic, neuroendocrine, and microbiota-derived influences that modulate intestinal immune responses in vivo, limiting its ability to fully replicate the complexity of the canine gastrointestinal environment. Pharmacokinetic aspects, including the low solubility, extensive metabolism, and limited bioavailability of many flavonoids, are also not represented in this model, necessitating cautious extrapolation of the applied concentrations to in vivo conditions [29]. Taken together, these limitations restrict direct conclusions regarding clinical efficacy in canine chronic inflammatory enteropathy and underscore the need for future studies incorporating multiple donor animals and in vivo validation.

Despite these limitations, these findings contribute to a growing body of evidence supporting the therapeutic potential of flavonoids in chronic enteropathies. Quercetin, luteolin, and GSOPs each demonstrated anti-inflammatory and antioxidant activity, reducing ROS generation and modulating cytokine output under LPS-induced stress. These multimodal actions highlight their potential as adjunctive nutraceutical strategies that may complement or, in some cases, reduce the reliance on long-term immunosuppressive or antibiotic therapies in canine patients.

## 5. Conclusions

In conclusion, the flavonoids examined in this study demonstrated protective anti-inflammatory and antioxidant effects in a canine duodenal explant model of LPS-induced inflammation. By modulating key oxidative and cytokine-mediated pathways, these compounds exhibited mechanistic potential to influence processes implicated in canine CIE. However, future studies incorporating multiple donor animals, as well as in vivo pharmacokinetic and efficacy investigations, will be essential to clarify the translational relevance of these observations in veterinary practice.

## Data Availability

The data are contained within the article. Further inquiries can be directed to the corresponding author.
